# Agreement between three noninvasive temperature monitoring devices during spinal anaesthesia for caesarean delivery: a prospective observational study

**DOI:** 10.1007/s10877-024-01154-1

**Published:** 2024-04-30

**Authors:** DO Vawda, Christopher King, L du Toit, RA Dyer, NJ Masuku, DG Bishop

**Affiliations:** 1 Department of Anaesthesia, School of Clinical Medicine, College of Health Sciences, Grey’s Hospital, University of KwaZulu-Natal, Pietermaritzburg, 3201 South Africa; 2 Department of Anesthesiology, Washington University School of Medicine in St Louis, MO, USA; 3 Department of Anaesthesia and Perioperative Medicine, University of Cape Town, Cape Town, South Africa

**Keywords:** Caesarean delivery, Hypothermia, Obstetric anaesthesia, Spinal anaesthesia, Thermoregulation, Temperature monitoring

## Abstract

Hypothermia during obstetric spinal anaesthesia is a common and important problem, yet temperature monitoring is often not performed due to the lack of a suitable, cost-effective monitor. This study aimed to compare a noninvasive core temperature monitor with two readily available peripheral temperature monitors during obstetric spinal anaesthesia. We undertook a prospective observational study including elective and emergency caesarean deliveries, to determine the agreement between affordable reusable surface temperature monitors (Welch Allyn SureTemp® Plus oral thermometer and the Braun 3-in-1 No Touch infrared thermometer) and the Dräger T-core© (using dual-sensor heat flux technology), in detecting thermoregulatory changes during obstetric spinal anaesthesia. Predetermined clinically relevant limits of agreement (LOA) were set at ± 0.5 °C. We included 166 patients in our analysis. Hypothermia (heat flux temperature < 36 °C) occurred in 67% (95% CI 49 to 78%). There was poor agreement between devices. In the Bland-Altman analysis, LOA for the heat flux monitor vs. oral thermometer were 1.8 °C (CI 1.7 to 2.0 °C; bias 0.5 °C), for heat flux monitor vs. infrared thermometer LOA were 2.3 °C (CI 2.1 to 2.4 °C; bias 0.4 °C) and for infrared vs. oral thermometer, LOA were 2.0 °C (CI 1.9 to 2.2 °C; bias 0.1 °C). Error grid analysis highlighted a large amount of clinical disagreement between methods. While monitoring of core temperature during obstetric spinal anaesthesia is clinically important, agreement between monitors was below clinically acceptable limits. Future research with gold-standard temperature monitors and exploration of causes of sensor divergence is needed.

**Supplementary Information:**

The online version contains supplementary material available at 10.1007/s10877-024-01154-1.

## Introduction

Hypothermia is a common and important problem in the perioperative period during both general and neuraxial anaesthesia [1]. Caesarean delivery (CD) is commonly performed under spinal anaesthesia (SA) due to its superior maternal and neonatal safety profile. However, SA is still associated with perioperative complications, including hypothermia. Hypothermia may lead to immune dysfunction, surgical site infections, adverse cardiac events, increased blood loss, and increased length of hospital stay [2–5]. Du Toit et al. showed that parturients undergoing obstetric SA develop perioperative hypothermia that persists into the recovery period, and thermal recovery may be delayed for several hours [6]. Despite this, in two surveys of practice monitoring of temperature under SA was not routinely performed by anaesthetists [7, 8]. This is likely due to the lack of an acceptable, noninvasive temperature monitoring method. Readily available noninvasive techniques tend to underestimate temperatures, particularly at lower core temperature values [5, 8].

Recent advances in noninvasive thermoregulatory monitoring include dual-sensor heat flux technology, delivering validated core temperature measurements [9, 10]. However, until recently this technology was not widely available. The single-use nature and cost associated with these monitors have precluded routine use in state hospitals in South Africa and in low-middle income settings. Affordable and cost-effective monitors such as oral thermometers and infrared thermometers are limited by concerns about reliability and validity of measurements in the perioperative setting.

We undertook a prospective observational study to determine the agreement between affordable reusable temperature monitors (an oral thermometer and a non-contact infrared thermometer) and a dual-sensor heat flux monitor, and between the oral and infrared thermometers, in detecting thermoregulatory changes during obstetric SA.

## Methods

We conducted a single centre prospective observational study of parturients undergoing obstetric SA at Harry Gwala Regional hospital in KwaZulu-Natal, South Africa. We collected additional data on patients participating in a separate study, which aimed to quantify the incidence and severity of perioperative hypothermia using a dual-sensor heat flux monitor (Dräger T-core©, Lübeck, Germany). In this study, we aimed to compare temperature measurements collected with this heat flux monitor, an oral thermometer (Welch Allyn SureTemp® Plus), and an infrared thermometer (Braun 3-in-1 No Touch). The study was approved by the University of KwaZulu-Natal Biomedical Research Ethics Committee (BREC/00003124/2021), and the Health Research Committee of the KwaZulu-Natal Department of Health (NHRD ref: KZ_202111_007). Informed, written consent was obtained in all participants.

### Patient selection

We recruited consecutive patients scheduled for elective or emergency CD under SA between 07h30 and 16h00 on normal working days (Monday to Friday, public holidays excluded). Patients undergoing CD after-hours, or on weekends and public holidays, were excluded due to the inability to collect data due to limited staff during these periods. This precluded collection of data at night when ambient temperatures are likely to be lower. We also excluded patients with age < 18 years, gestational age < 28 weeks, a history suggestive of symptomatic thyroid disease, or no consent. Participants converted to general anaesthesia for any reason were excluded from analysis.

### Technology

Temperature measurement was obtained using a heat flux monitor. This uses a self-adhesive sensor placed on the participant’s forehead, containing two temperature sensors separated by an insulating layer (dual-sensor heat flux technology) [11]. One sensor measures the temperature at the surface of the skin, and the other measures the flow of heat to the environment [11]. Following a short warmup time, the sensor calculates core body temperature continuously [11]. The technology has been shown to have a high degree of accuracy and precision in other settings, comparable with that of the thermistor of the Swan-Ganz catheter, and oesophageal and bladder temperature probes [12]. Temperature measurements were also taken using an oral thermometer, and an infrared thermometer.

### Procedures

All temperature measurements were taken by the anaesthesia providers, and departmental training in the use of the three devices was conducted prior to commencement of the study. The heat flux sensor was applied on the right side of the participant’s forehead at the same time as the other routine monitors were applied, prior to spinal injection. Oral temperatures were taken using a new disposable sheath for each patient. Surface temperatures were also measured, without direct patient contact, using an infrared thermometer (held at 3–5 cm from the centre of the forehead, with the skin cleaned and dried). Temperature data was collected at four time points for each device. A baseline temperature (T0) was obtained for each device at the time of initiation of SA, followed by temperatures at 10-minute intervals for a total of 30 min (a total of 4 readings). Secondary maternal outcomes were collected for the parent study. Ambient temperature at the time of SA was measured by a fixed, wall-mounted, digital thermometer in the operating theatre.

### Conduct of anaesthesia

Normal standards applicable to obstetric anaesthesia at Harry Gwala Regional Hospital were followed. Interns and trainee anaesthetists administered anaesthesia under the supervision of an experienced anaesthetist. Standard SA dosing was 9 mg 0.5% hyperbaric bupivacaine and 10 µg fentanyl injected at the L3/4 interspace, using a 25G atraumatic spinal needle. Hypotension was treated with a bolus of phenylephrine or ephedrine, aiming to maintain systolic blood pressure at ≥ 90% of the baseline systolic blood pressure measured preoperatively in theatre. Refractory hypotension was treated with a phenylephrine infusion. Oxytocin 2.5 international units (IU) was given intravenously after delivery, with a further 7.5 IU as an infusion. There was no routine prewarming of participants. The unit protocol is to use warmed intravenous fluid from a fluid warmer set at 43 °C, and a forced air warmer (3 M™ Bair Hugger™ Upper Body Blanket, Maplewood, Minnesota, United States of America) if available. All blood and blood products infused were warmed using a blood/fluid warming system set at 38 °C. Data was recorded on a paper-based case report form by one member of the anaesthesia team, consisting of an experienced anaesthetist and a trainee. Oral and infrared thermometer readings were recorded directly onto the case report form as the measurements were taken. Heat flux temperatures were recorded from the Dräger anaesthesia workstation’s trend table at the appropriate time intervals. All case report forms were stored in a secure area at the end of each day by one of the investigators.

### Statistical analysis

We used the Strengthening the Reporting of Observational Studies in Epidemiology (STROBE) statement guidelines to report our findings [13]. The baseline characteristics of the included participants were reported as median (interquartile range [IQR]); and count (percent) for categorical variables. To quantify the between-device agreement of repeated measurements of temperature, we used a modified Bland-Altman method based on variance components [14, 15]. Briefly, the traditional Bland-Altman method assumes that all observations are independent [16], and, when naively applied to repeated measures on the same participant, underestimates the uncertainty and over-emphasises data from individuals with longer observation periods. The repeated measures model assumed a data-generating mechanism with a person-specific starting temperature at the time of SA, an average change at each time point, a device-specific bias, and independent variation representing measurement noise and person-specific trends. Because of the relatively short series (4 points per person), parametric person-specific trends were not included. Based on the methods of Myles [17], we fitted a linear mixed effects model, and calculated the probability of future measurement agreeing at clinically relevant margins. We prespecified ± 0.5 °C as a target limit of agreement (LOA), as this was clinically relevant and in keeping with other studies in the field [18]. Bootstrap 95% confidence intervals (CIs) for the LOA were calculated using the parametric method (not conditional upon random effects) with percentile limits. We also report correlations without any repeated measures adjustment, but using non-parametric bootstrap CIs with the percentile method grouping at the participant level. The calculations for all comparisons used all time points with available data for both sensors. To characterise the clinical relevance of disagreements between sensors, an error grid was overlaid on the results [19, 20].

Because large changes exhibited by some heat flux sensors in the first 10 min of data collection were thought to be due to inadequate time for equilibrium, we conducted a sensitivity analysis without the t = 0 data and reported correlations for each time point separately. Figures in the main analysis were generated using all data and excluding values which were clearly erroneous (≤ 34 °C). For all analyses a *p-*value of < 0.05 defined statistical significance.

An initial sample size calculation, using an effect size difference of 0.5, an α value of 0.05 and 80% power, showed that 64 participants were required. However, since we were collecting data on a larger sample for the parent study, we aimed to collect data on all patients enrolled in that study, allowing a narrower standard deviation of the differences between measurements.

Analyses were performed using R version 4.1.2 (R Foundation for Statistical Computing, Vienna, Austria). A container replicating the environment and code for the analysis is located at (https://github.com/cryanking/temp_agreement).

## Results

Data collection occurred from 02 August 2021 to 28 October 2021. During this period, 863 CDs were performed. Of these, 468 were ineligible for recruitment, due to method of anaesthesia or time of CD. Of the remaining 395 CDs, we recruited 180 patients who fulfilled eligibility criteria. Consecutive patients were recruited based on availability of monitors. No patient refused consent. Fourteen recruited participants were subsequently excluded from analysis because they did not meet study criteria (eight were < 18 years old; four cases were converted to general anaesthesia; two participants had a gestational age < 28 weeks). Of the 166 included patients, 109 (66%) were elective CD and 57 (34%) were urgent or emergency CD, including 21 (13%) patients in active labour.

The median age of participants was 29 years (IQR 24–34) and median gestational age was 39 weeks (IQR 38–40). Forced air warming (FAW) devices were used in 95% of participants, and warmed intravenous fluids in 85%. Median ambient theatre temperature was 19 °C (IQR 18–21). Ignoring implausible measurements of less than 34 degrees, hypothermia was detected by heat flux in the first 30 min in 67% of participants (95% CI 59 to 74%), by IR in 40% (95% CI 32 to 48%), and by oral measurement in 10% (95% CI 6 to 16%). Median blood loss was 550 ml (IQR 500–700 ml). Nine participants had estimated blood loss ≥ 1000 ml. Intraoperative shivering was experienced by 34 (21%) participants. Vomiting occurred in 20/166 patients. Three neonates required direct admission to the neonatal intensive care unit, and two neonates required cardiopulmonary resuscitation. All included patients had temperature measurements performed using the heat flux-, infrared-, and oral thermometer.

**Fig. 1:**
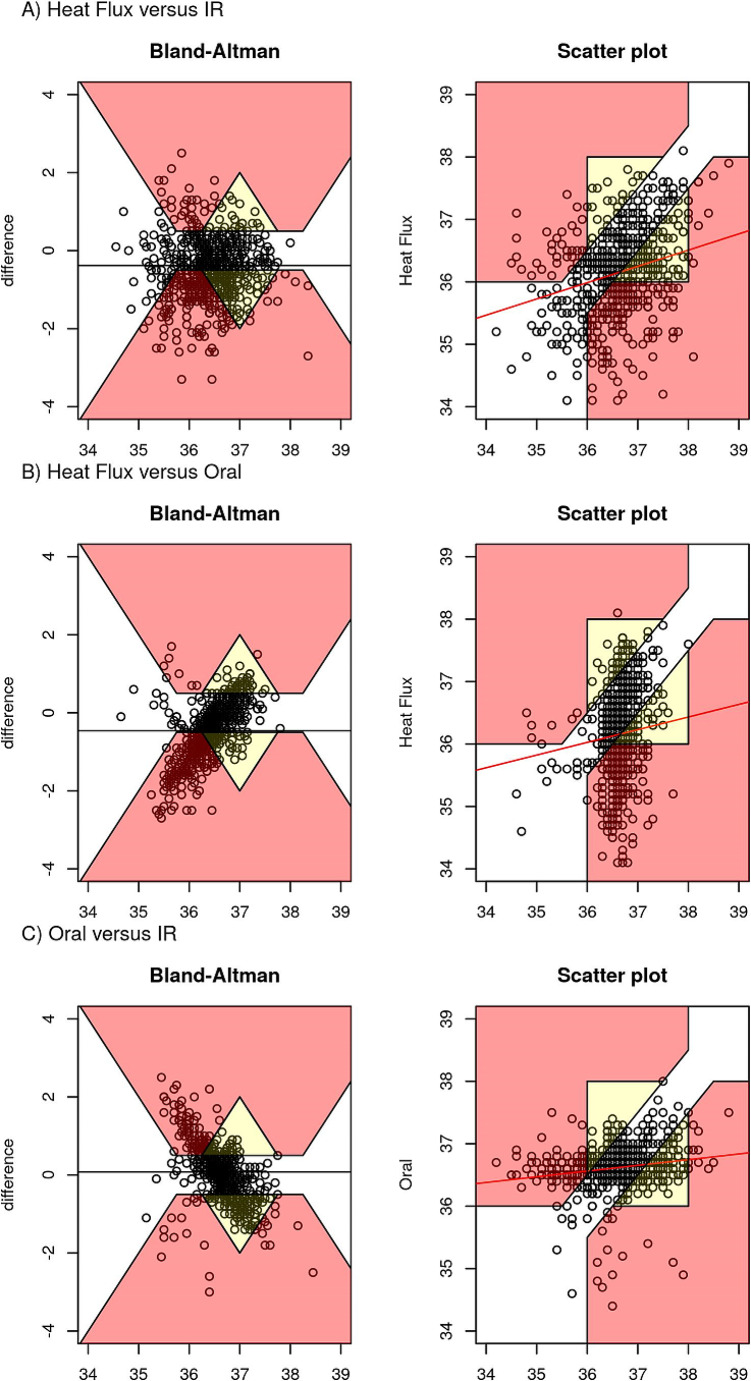
Error Grid analysis, with modified Bland Altman analysis for repeated measures (left) and scatter plots (right) between different temperature monitors, excluding erroneous measurements (≤ 34 °C). *Heat flux: Dräger T-core©; IR: Braun 3-in-1 No Touch infrared thermometer; Oral: Welch Allyn SureTemp® Plus oral thermometer*

Table 1 reports the Bland Altman analyses for the three comparisons, with summary statistics of the agreement between sensors. Figure 1 reports the corresponding Bland Altman plots and scatter plots for between-device correlation with error grids overlaid. Figure S1 in the appendix report the same plots but without the error grids. The mean LOA for all comparisons is outside the predetermined clinically relevant LOA of ± 0.5 °C.

**Table 1:** Bias and limits of agreement between devices

Monitor Comparison	Duration	Mean Bias	Standard error of the bias	Mean LOA	Lower boundary of the LOA	Upper boundary of the LOA
Heat flux vs. Oral	T0 – T30	-0.5	0.1	1.8	1.7	2.0
Heat flux vs. IR	T0 – T30	-0.4	0.1	2.3	2.1	2.4
IR vs. Oral	T0 – T30	0.1	0.1	2.1	1.9	2.2

*Heat flux: Dräger T-core©; IR: Braun 3-in-1 No Touch infrared thermometer; Oral: Welch Allyn SureTemp® Plus oral thermometer; LOA: limits of agreement. The mean bias is the mean difference, averaged over all points at which both measures are available. The standard error of the bias is the estimated standard error of the mean difference accounting for repeated measures. The LOA values for each comparison account for repeated measures*

*Comparison A: Heat flux monitor versus oral thermometer.* The oral thermometer readings were on average 0.5 °C higher, and 42% of readings were within the prespecified 0.5 °C LOA. Overall correlation between the readings was low (correlation coefficient 0.2).

*Comparison B: Heat flux monitor versus infrared thermometer*. The infrared thermometer readings were on average 0.4 °C higher, and 38% of readings were within the prespecified 0.5 °C LOA. Overall correlation between the readings was low (correlation coefficient 0.4).

*Comparison C: Oral thermometer versus infrared thermometer*. The two monitors showed similar mean values (bias 0.1 °C). The overall correlation (0.2) and fraction of readings within acceptable LOA (52%) were low.

The calculated limits of agreement (95% prediction intervals for differences in future measurements) appeared somewhat conservative on the data, covering 98% of observed differences.

To illustrate the clinical relevance of differences between monitors, we overlay an error grid [19] using the same criteria as [20]: if two monitors are within 0.5 degrees of each other (or the average in the BA plot) then the error is clinically negligible, Fig. 1. If two monitors yield the same interpretation for clinical action (both less than 36.0, or both greater than 38.0) then the error is clinically negligible. If the monitors disagree by greater than 0.5 degrees, but neither indicates hypo or hyperthermia, then the error is relevant but not clinically actionable (yellow). If one monitor suggests clinical actions and the other does not, the difference is clinically significant (red). Ignoring implausible measurements below 34 degrees centigrade, hypothermia was detected by heat flux in the first 30 min in 67% of participants (95% CI 59 to 74%), by IR in 40% (32 to 48%) and oral in 10% (6–16%).

There were inconsistent mean differences (slope in the Bland-Altman plots in Fig. S1) for comparisons B) heat flux versus oral thermometer and comparison C) oral thermometer versus infrared thermometer. These slopes were seen because, on average, oral temperature remained comparatively constant across a range of measured heat flux and infrared temperatures. In other words, at lower mean temperatures (Y-axes), oral temperature was higher than heat flux and infrared temperatures and at higher mean temperatures, oral temperature was lower than heat flux and infrared temperatures.

Table S1 in the appendix, reports two sensitivity analyses, (i) inclusion of extreme (erroneous) temperatures (≤ 34 °C) from the analysis and (ii) excluding timepoint T0 from the analysis, and compares these sensitivity analyses with the main analysis which includes timepoint T0 and excludes extreme temperatures (≤ 34 °C). Figure S2 in the appendix, reports the corresponding Bland Altman plots and scatter plots for between-device correlation for the first sensitivity analysis (inclusion of temperatures ≤ 34 °C). Table S2 reports the correlation and limits of agreement between devices at different time points. There was no discernible change in the measures of agreement over time.

## Discussion

Hypothermia is associated with serious perioperative complications. Patients who have obstetric surgery under general or neuraxial anaesthesia should have intraoperative temperature monitoring, in accordance with guideline recommendations by national bodies and anaesthesia societies [21–23]. The lack of acceptable and affordable noninvasive core temperature monitors are limiting factors [24]. Our study aimed to determine the agreement between three noninvasive temperature monitors that could be used to monitor temperature changes in parturients undergoing SA for CD, in order to accurately identify patients at risk for complications of hypothermia. There was poor agreement between the three devices.

Maintenance of body temperature forms an important part of normal homeostatic function in humans. During the perioperative period, homeostatic function can be disturbed, putting patients at increased risk of developing significant thermoregulatory disturbances. Thermoregulatory disturbance is defined as an increase or decrease in core temperature to < 36.0**°**C or > 38.0**°**C, or an increase or decrease in core temperature by > 1**°**C from baseline [6]. These disturbances are seen during both neuraxial and general anaesthesia [4, 6]. During neuraxial anaesthesia, central to peripheral redistribution of heat can be profound, and is associated with the inability to fully compensate for a decrease in core temperature by the production of heat energy through shivering [4, 25]. Furthermore, compensation may be impaired for a significant period of time, with recovery of core temperature only occurring hours later [6]. Such a decrease of core temperature, coupled with delayed recovery, may influence the physiology of cardiac and renal function, haematology, and the immune system [2, 3]. These consequences are of particular concern in obstetric anaesthesia because SA is the commonest anaesthetic technique employed, and the impact of persistent postoperative hypothermia has not been adequately studied in this population.

There are several noninvasive methods of measuring temperature. These include forehead skin temperature using a liquid crystal thermometer, temporal artery temperature monitors, infrared skin surface thermometers, oral thermometers, and heat flux temperature monitoring systems. However, studies assessing the accuracy of available noninvasive temperature monitors suggest that most are prone to errors in measurement [24]. Torossian et al. have identified acceptable invasive and noninvasive core temperature monitors. Invasive core temperature monitors include the pulmonary artery catheter, nasopharyngeal, oesophageal and bladder thermometers. The pulmonary artery catheter is widely considered to be the gold standard or reference temperature monitor; however, its use is not routine due to risk of complications. Noninvasive core temperature monitors include oral temperature measurement, forehead zero heat flux or dual sensor thermometers, and tympanic membrane contact thermometers. Infrared thermometers, including skin surface, tympanic membrane and temporal artery, are not considered suitable for intraoperative usage [24].

Gomez-Romero et al. showed that heat flux temperature monitoring systems, such as the Dräger T-core™ and the 3 M™ SpotOn™ system, have clinically acceptable LOA compared to the pulmonary artery catheter in temperature measurement [11]. In their work, Gomez-Romero et al. emphasise a trend towards more dispersion with the Dräger T-core™ system, although not reaching statistical significance. The Dräger T-core™ and the 3 M™ SpotOn™ zero heat flux temperature monitoring systems have fundamental design differences. The Dräger T-core™ system, although considered a zero heat flux monitor, utilises a non-zero heat flux method. Here temperatures are measured across two points of unequal, but known thermal resistances [10].

The 3 M™ SpotOn™ temperature monitor employs an insulating cover within which a single thermopile is embedded. With time the transfer of heat from the patient into the insulated area will cease, whereafter any fluctuations in temperature represent a change in core temperature. This method of detecting temperature changes on a surface is known as isothermal channelling [26]. However, both monitors have been validated for clinical use in multiple clinical settings [9, 10]. For perioperative clinical use, heat flux thermometry seems ideal, as it is noninvasive, comfortable, and provides continuous core temperature measurement [11, 27]. Cost is the major limitation to the use of this method, due to the fact that the probes are single-use. This makes its use problematic in resource-limited settings.

Oral thermometers use thermocouples or thermistors to measure temperature. Lawson et al. found that the Welch Allyn SureTemp® Plus thermometer agreed with pulmonary artery catheter temperatures within clinically acceptable limits (± 0.5 °C), with a bias of 0.09 °C (CI -0.75 to 0.93 °C) in an intensive care setting [28]. Limitations are that oral thermometers have not been validated in the perioperative environment, they do not provide continuous measurement, and can be influenced by varying environmental temperatures. Benefits of the use of oral thermometers are that they are reusable, and noninvasive, having significant impact on cost effectiveness. Therefore, we included the oral thermometer as a potentially feasible tool for perioperative care of the obstetric patient under SA. However, our findings demonstrated poor agreement between monitors, suggesting that further studies are required to validate the use of oral thermometers in perioperative obstetric care.

Infrared thermometers operate through the use of a thermopile which detects infrared energy released from any surface. Tympanic membrane measurements have been shown to approximate core temperatures accurately, but are limited by discomfort for patients and training requirements for accurate readings [29]. Recently, infrared temperature monitoring of other body sites has increased, particularly as a screening tool in the Covid-19 pandemic, although they do not provide a valid measurement of core temperature. The inexpensive nature of infrared thermometers makes them an attractive tool for thermoregulatory monitoring, but perioperative use is limited, likely because poor agreement has been demonstrated with other noninvasive core temperature monitors [18]. Due to the fact that this monitor is reusable, economical, and there is avoidance of unnecessary patient contact, we included it as a device for further investigation. Our conclusions were similar to those of Holder et al., with poor agreement on Bland Altman analysis [18]. We also noted that the infrared thermometer measured 0.4 °C higher than the heat flux monitor and that this was consistent across all temperature ranges.

Overall our study found poor agreement in all comparisons between monitors, with unacceptably wide LOA. This indicates that these devices cannot be used interchangeably. However, the three monitors we compared measure temperatures at different sites, and we did not expect deep brain temperature, oral temperature and forehead skin temperature to be identical. The body sites are expected to have different temperatures and these differences are expected to change depending on the core to peripheral temperature gradient. It is also noteworthy that both the oral and infrared thermometers recorded higher mean temperatures than the heat flux technology at low mean temperatures. Healthcare quality may be assessed using process measures such as perioperative hypothermia, and the choice of device might therefore affect measured compliance with absolute temperature targets.

We included both elective and emergency CD in our study, with 21 (13%) of patients being in active labour. Available data has tended to focus on the elective CD population, although a comparable recent study included emergency CD [30]. There are physiological differences in thermoregulation between labouring and non-labouring parturients, with activation of thermogenesis in the former group. The increased temperature in some women during labour may also be associated with increased body mass index and duration of rupture of membranes [31]. These women may therefore begin the perioperative period with slightly higher baseline temperatures and a more substantial temperature buffer. The heterogeneity of our population may improve the generalisability of our results.

There were some limitations to our study. The inclusion of emergency CD may necessitate multiple actions from the anaesthetist in a short space of time. It is therefore possible that error may occur in technique for temperature measurement. However, the inclusion of both elective and emergency patients is also an important strength, given the physiological differences discussed above. We limited data collection to working hours, when additional junior staff were available to assist with data collection, to mitigate this concern. It is also possible that the placement of the heat flux sensor may have influenced the infrared temperature measurements if the latter were taken immediately next to the heat flux sensor. However, our conditions represent real-world settings and are thus applicable to similar environments. Testing devices in the clinical environment is an important part of validation.

Strengths of our study include that we analysed devices used by anaesthetists in a real-world setting, making our findings generalisable. Our results suggest that it is not advisable to use these devices interchangeably, and while trends may give attending anaesthetists valuable information, the accuracy of the specific measurement could be questioned. The importance of avoiding hypothermia in the obstetric population, coupled with the high incidence of hypothermia we found, suggest that further research into accurate temperature monitoring in this population is required.

## Conclusion

In this study, three noninvasive thermometers showed poor agreement between devices. Although the oral and infrared monitors are used outside the perioperative setting for intermittent readings, their acceptability in perioperative medicine was not demonstrated on Bland-Altman analysis. Further work in this field is required, using gold standard temperature monitors and analysis into the causes of sensor divergence between different modalities, to develop the optimal temperature monitor for the awake obstetric patient receiving SA for CD.

## Electronic supplementary material

Below is the link to the electronic supplementary material.

Supplementary Material 1

## Data Availability

A container replicating the environment and code for the analysis is located at (https://github.com/cryanking/temp_agreement).
