# Supplementary material for: Agreement between three noninvasive temperature monitoring devices during spinal anaesthesia for caesarean delivery: a prospective observational study
Source: J Clin Monit Comput. 2024 Apr 30;38(5):1199–207. doi: 10.1007/s10877-024-01154-1 (PMC11427619; doi:10.1007/s10877-024-01154-1)
Supplement: Supplementary file 1 — Supplementary Material 1 [file 10877_2024_1154_MOESM1_ESM.docx]

**Title Page: Appendix**

**Title:** Agreement between three noninvasive temperature monitoring devices during spinal anaesthesia for caesarean delivery: a prospective observational study.

1. DO Vawda^1^ [https://orcid.org/0009-0002-6401-0489]
2. Christopher King^2^ [https://orcid.org/0000-0002-4574-8616]
3. L du Toit ^2,3^ [https://orcid.org/0000-0003-0146-4002]
4. RA Dyer^3^ [https://orcid.org/0000-0002-8165-9098]
5. NJ Masuku^1^ [https://orcid.org/0000-0001-6250-2389]
6. DG Bishop^1^ [http://orcid.org/0000-0001-9861-3646]

**Corresponding Author:**

DG Bishop

[davidgbishop@gmail.com](mailto:davidgbishop@gmail.com)

Anaesthetic Department

Grey’s Hospital

Town Bush Road

Pietermaritzburg

KwaZulu-Natal

South Africa

3201

^1^ Department of Anaesthesia, School of Clinical Medicine, College of Health Sciences, University of KwaZulu-Natal, Pietermaritzburg, South Africa

^2^ Department of Anesthesiology, Washington University School of Medicine in St Louis, MO, USA

^3^ Department of Anaesthesia and Perioperative Medicine, University of Cape Town, Cape Town, South Africa

**Appendix**

***Supplementary methods:***

Bland-Altman analysis [1] derives 95% limits on the difference between two measurements assuming that the underlying quantity being measured is the same. Bland and Altman subsequently extended this work to account for repeated measures over time on individual participants in and ANOVA framework by taking differences between methods and a random effect for timepoint [2]. Myles and Cui further extended this to a mixed linear model allowing us to detrend for confounders which are not balanced between participants; in this case, time after spinal block [3]. Parker and colleagues [4] review alternatives and developments for agreement quantification with explicit formulas.

Following Myles and Parker, we adopt a model in which two instruments are simultaneously compared using a mixed linear model with a fixed effect for instruments, fixed effect for timepoint (t) using a centered representation, a random effect for interaction with participant (i), and true noise plus person-specific drift,

E[ y_1ti - y_2ti] = mu_t + gamma_i + epsilon_it ,

fitted by maximum likelihood.

We are seeking a quantity c which bounds the difference between two future measurements (delta) with probability 0.95, assuming that known bias (mu) can be eliminated. Let tau = var(gamma), sigma = var(epsilon)

Pr(abs(delta) < c ) = 0.95

implies

Pr(delta^2 < c^2 ) = 0.95

which expands to

Pr(N(0, tau + sigma) ^2 < c^2 ) = 0.95

To account for the uncertainty in the estimated tau and sigma, we model the MLE of the log of total variance as normal with mean log(tau + sigma) and standard deviation k. We estimate k by bootstrap.

Pr(ChiSq(1)* exp(log( tau + sigma) + z*k) < c^2 ) = 0.95

Implies

Pr(ChiSq(1) < c^2 * exp(-log( tau + sigma) + z*k) ) = 0.95

And we use the law of iterated expectation to write this as a function of two random variables Chi and z

E[E[Chi < c^2 * \hat(tau + sigma)^-1 exp(z)^k) )] ] = 0.95

Using the chi square distribution function with one degree of freedom (pchisq)

E[pchisq ( lambda^2 * exp(z)^k) ) ] = 0.95

where lambda = c sqrt(\hat( tau + sigma)^-1 ), which is easily solved for lambda using monte carlo over z.

Finally, to obtain the estimate of z, we multiply

\hat (c) = \hat(lambda) * sqrt(\hat( tau + sigma) )

with bootstrap 95% limits on \hat(tau + sigma).

To report the uncertainty in the point estimate of the bias (mu), we use the method of Kenward and Roger [5], and graphically display mu +- c

Table S1 displays summary statistics of the agreement between sensors. Since the heat flux monitor requires several minutes before equilibrium is reached, we aimed to exclude the possibility that inadequate warmup time influenced our results. This sensitivity analysis was achieved by comparing the analysis for the entire study period (thirty minutes, time 0 – time 30) with an analysis excluding the baseline measure at time 0 (time 10 -time 30). We further included in Table S1 an analysis removing all temperature points < 34°C for the entire time period. This resulted in 15 data points removed for the heat flux, 16 for the infrared and 4 for the oral temperature monitors. The LOA estimates were improved following this analysis, but the qualitative interpretation remained similar.

Table S2 shows the analysis at each individual time point. LOA is the estimated limit of agreement, while LOA success refers to the fraction of measurements that are within the specified clinical LOA. Figure S1 shows the Bland Altman analysis that was reported in the main paper, and Figure S2 shows a similar analysis, but including values < 34°C.

**Table S1. Bias and limits of agreement between devices.**

| **Monitor**  **comparison** | **Time period** | **Mean bias** | **Standard error of the bias** | **Mean level of agreement** | **Lower bound of the LOA** | **Upper bound of the LOA** |
| --- | --- | --- | --- | --- | --- | --- |
| **Heat Flux vs IR** | T0 – T30 | -0.4 | 0.1 | 2.3 | 2.1 | 2.4 |
|  | T10 – T30 | -0.3 | 0.1 | 2.2 | 2.1 | 2.4 |
|  | T0 – T30 (filter < 34°C) | -0.5 | .05 | 1.6 | 1.5 | 1.7 |
| **Heat Flux vs Oral** | T0 – T30 | -0.5 | 0.1 | 1.8 | 1.7 | 2.0 |
|  | T10 – T30 | -0.4 | 0.1 | 1.7 | 1.5 | 1.8 |
|  | T0 – T30 (filter < 34°C) | -0.5 | 0.05 | 1.4 | 1.4 | 1.6 |
| **IR vs Oral** | T0 – T30 | 0.1 | 0.1 | 2.0 | 1.9 | 2.2 |
|  | T10 – T30 | 0.1 | 0.1 | 2.0 | 1.9 | 2.2 |
|  | T0 – T30 (filter < 34°C) | -0.01 | 0.04 | 1.4 | 1.3 | 1.5 |

*Heat flux: Dräger T-core©; IR: Braun 3-in-1 No Touch infrared thermometer; Oral: Welch Allyn SureTemp® Plus oral thermometer; LOA: limits of agreement. The mean bias is the mean difference, averaged over all points at which both measurements are available. The standard error of the bias is the estimated standard error of the mean difference accounting for repeated measures. The LOA values for each comparison account for repeated measures.*

**Table S2. The correlation and limits of agreement values between devices at different time points.**

| **Variable 1** | **Variable 2** | **Time point** | **Correlation** | **Lower bound of the**  **LOA** | **Upper bound of the**  **LOA** | **Mean LOA**  **Success** | **Lower bound of LOA**  **Success** | **Upper bound of LOA**  **Success** |
| --- | --- | --- | --- | --- | --- | --- | --- | --- |
| Heat Flux | Oral | All | 0.23 | 0.14 | 0.3 | 0.42 | 0.36 | 0.46 |
| Heat Flux | Oral | Time 0  excluded | 0.24 | 0.13 | 0.34 | 0.43 | 0.37 | 0.48 |
| Heat Flux | Oral | 0 | 0.21 | 0.08 | 0.32 | 0.39 | 0.31 | 0.46 |
| Heat Flux | Oral | 10 | 0.11 | 0.36 | 0.44 | 0.37 | 0.52 | 0.24 |
| Heat Flux | Oral | 20 | 0.04 | 0.36 | 0.44 | 0.36 | 0.52 | 0.21 |
| Heat Flux | Oral | 30 | 0.12 | 0.35 | 0.4 | 0.33 | 0.48 | 0.24 |
| Heat Flux | IR | All | 0.35 | 0.24 | 0.46 | 0.38 | 0.33 | 0.43 |
| Heat Flux | IR | Time 0  excluded | 0.37 | 0.24 | 0.49 | 0.38 | 0.32 | 0.43 |
| Heat Flux | IR | 0 | 0.31 | 0.18 | 0.44 | 0.38 | 0.3 | 0.46 |
| Heat Flux | IR | 10 | 0.34 | 0.18 | 0.5 | 0.4 | 0.32 | 0.48 |
| Heat Flux | IR | 20 | 0.37 | 0.2 | 0.52 | 0.4 | 0.32 | 0.47 |
| Heat Flux | IR | 30 | 0.24 | 0.12 | 0.35 | 0.4 | 0.33 | 0.48 |
| Oral | IR | All | 0.23 | 0.14 | 0.32 | 0.52 | 0.48 | 0.57 |
| Oral | IR | Time 0  excluded | 0.26 | 0.18 | 0.35 | 0.54 | 0.48 | 0.59 |
| Oral | IR | 0 | 0.14 | -0.04 | 0.32 | 0.48 | 0.4 | 0.56 |
| Oral | IR | 10 | 0.28 | 0.15 | 0.41 | 0.53 | 0.46 | 0.6 |
| Oral | IR | 20 | 0.25 | 0.16 | 0.36 | 0.54 | 0.47 | 0.62 |
| Oral | IR | 30 | 0.26 | 0.1 | 0.41 | 0.54 | 0.46 | 0.61 |

*Heat flux: Dräger T-core©; IR: Braun 3-in-1 No Touch infrared thermometer; Oral: Welch Allyn SureTemp® Plus oral thermometer; LOA: limits of agreement.*


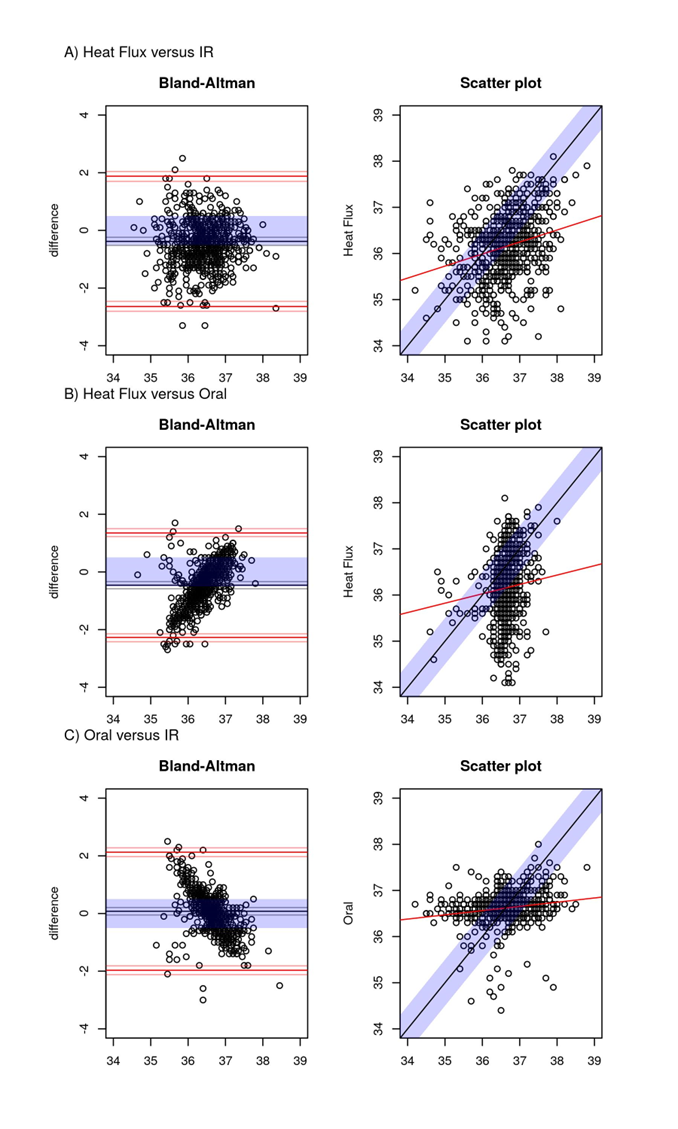


**Figure S1. Modified Bland Altman analysis for repeated measures (left) and scatter plots (right) between different temperature monitors, excluding erroneous measurements (≤ 34°C).**

**Left: Bland Altman plot:** X-axis: mean temperature for the 2 sensors. Y-axis: bias. Black horizontal line: mean bias, with 95% CI. Red lines: calculated limits of agreement (mean ± 2 SD). blue box: 0.5°C, = clinically acceptable limits of agreement.

**Right**: **Scatter plot.** Black line: perfect agreement. Purple box: 0.5°C error limit. Red line: linear mixed model regression line.

*(Heat flux monitor: Dräger Tcore*©*, oral thermometer:* *Welch Allyn SureTemp® Plus, Infrared monitor: R:* *Braun 3-in-1 No Touch)*


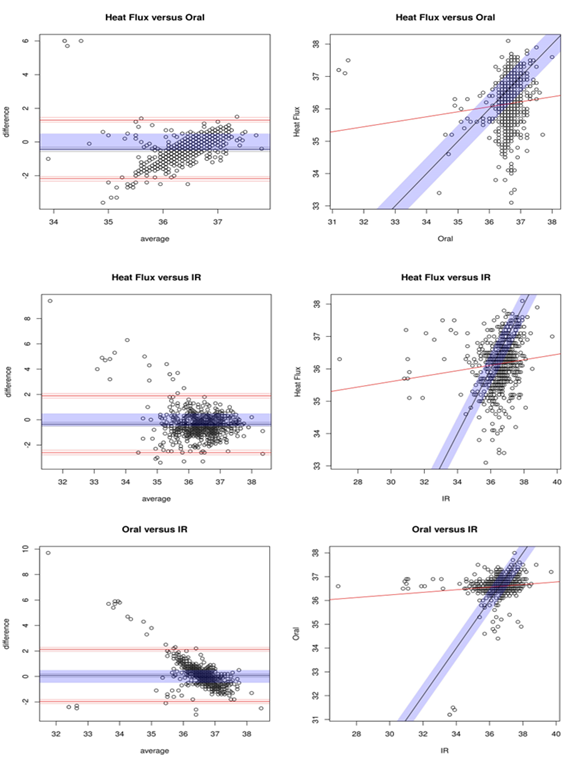


**Figure S2. Modified Bland Altman analysis for repeated measures (left) and scatter plots (right) between different temperature monitors, including erroneous measurements** **(≤ 34°C).**

**Left: Bland Altman plot.** X-axis: mean of sensor values. Y-axis: difference between sensor values (bias). Black horizontal line: observed mean bias between sensors, and 95% CI. Red lines: calculated limits of agreement, with 95% CI. Purple box: 0.5°C, = clinically acceptable limit of agreement.

**Right**: **Scatter plot.** Black line: perfect agreement. Purple box: 0.5°C error limit. Red line: linear mixed model regression line.

*(Heat flux monitor: Dräger Tcore*©*, oral thermometer:* *Welch Allyn SureTemp® Plus, IR:* *Braun 3-in-1 No Touch)*

**References**

1. Bland JM, Altman DG. Statistical Methods for Assessing Agreement between Two Methods of Clinical Measurement. The Lancet. 1986;327(8476):307-310. <https://doi.org/10.1016/s0140-6736(86)90837-8>.

2. Bland JM, Altman DG. Measuring agreement in method comparison studies. Stat Methods Med Res. 1999;8(2):135-160. <https://doi.org/10.1177/096228029900800204>.

3. Myles PS, Cui J. Using the Bland-Altman method to measure agreement with repeated measures. Br J Anaesth. 2007;99(3):309-311. <https://doi.org/10.1093/bja/aem214>.

4. Parker RA, Scott C, Inacio V, Stevens NT. Using multiple agreement methods for continuous repeated measures data: a tutorial for practitioners. BMC Med Res Methodol. 2020;20(1):154. <https://doi.org/10.1186/s12874-020-01022-x>.

5. Kenward MG, Roger JH. Small Sample Inference for Fixed Effects from Restricted Maximum Likelihood. Biometrics. 1997;53(3):983. <https://doi.org/10.2307/2533558>.
